# Isomers Identification of 2-hydroxyglutarate acid disodium salt (2HG) by Terahertz Time-domain Spectroscopy

**DOI:** 10.1038/s41598-017-11527-z

**Published:** 2017-09-22

**Authors:** Wanqing Chen, Yan Peng, Xiankai Jiang, Jiayu Zhao, Hongwei Zhao, Yiming Zhu

**Affiliations:** 1 Shanghai Key Lab of Modern Optical System, University of Shanghai for Science and Technology, No. 516, Jungong Road, 200093 Shanghai, China; 2 Shanghai Institute of Applied Physics, Chinese Academy of Sciences, 201800 Shanghai, China

## Abstract

2-Hydroxyglutaric acid disodium salt (2HG) is a unique biomarker existing in glioma, which can be used for recognizing cancer development stage and identifying the boundary between the ordinary tissue and cancer tissue. However, the most efficient detection method for 2HG now is Magnetic Resonance Spectroscopy (MRS), whose testing time is at least twenty minutes and the variability of 2HG (continuous synthesis and decomposition) determines it cannot be used as the real-time image in medical surgery. In this paper, by using the Terahertz Time-domain Spectroscopy (THz-TDS) System, we investigate the vibration spectra of 2HG isomers and further distinguish their physical properties by using Density Functional Theory. The differences between isomers are mainly attributed to the proton transfer inside the carbon chain. These results indicate that terahertz technology can identify the isomers of 2HG accurate and fast, which has important significance for the further investigation of glioma and clinical surgery.

## Introduction

Glioma is one of the most common central nervous system tumors, but only few type of glioma can be ‘cured’ during lifetime. Most glioma are easy to relapse even after operation, radiotherapy and chemotherapy, result in unfavorable prognosis. At present, the most noninvasive and reliable diagnosis method of glioma is magnetic resonance imaging (MRI). Besides, magnetic resonance spectroscopy (MRS) is a novel technique for more accurate diagnosis recently^1–3^. However, the boundary between tumor and normal tissue is usually unclear, making it difficult for surgeons to remove the tumor completely. Neuronavigation is a common method for real-time tumor location. However, brain shift after opening the dura will affect doctor’s judgment and then limit the accuracy of MRS navigation during surgery^4^.

Recently, some research groups found a unique biomarker——2HG in glioma^5–8^, which is over-accumulated during the process of lesion: Mutations in isocitrate dehydrogenase 1 and 2 (IDH1, IDH2) are widespread in IDH-mutated glioma patients. These mutations make IDH1 or IDH2 lose the function of clear metabolites (2HG), resulting the accumulation of 2HG within the tumor. In the area of medicine, as comparing with the change of glutamine and glutamate levels in the mutant glioma, significant differences in 2HG level are found between the low-grade gliomas [World Health Organization (WHO) grades II and III] and primary glioblastomas^9^. Therefore, 2HG can be used as a specific Magnetic Resonance Biomarker, monitoring IDH1 and IDH2, tracking glioma state and identifying the boundary between tumor and normal tissue. On the other side, it is found that pure 2HG does not exist in human body, it can only exist with the form of ionic compounds by combining with some metal ions in bodies such as the isomer of L-2-Hydroxyglutaric acid disodium salt (L-2HG) and D-2-Hydroxyglutaric acid disodium salt (D-2HG). In glioma, 2HG is mixture of L-2HG and D-2HG, whose ratio and contents affect the medical diagnosis between glioma (the content of L-2HG is more than that of D-2HG) and the acute myelogenous leukemia ((the content of D-2HG is more than that of L-2HG))^10,11^. However, the existing MRS technology cannot identify them effectively. Therefore, a new real-time method for identifying 2HG isomers and accurate locating the tumor region during surgery is urgently needed.

Terahertz (THz) spectroscopy has been widely known due to its high penetrability, low ionization energy, fingerprint spectrum and coherence. In recent decades, THz spectroscopic technique has proved its effectiveness in detection of skin, breast, tongue, liver, colon tumors^12–14^ and also the mixtures identification^15^. Here, in this paper, we expect to realize the spectral detection and the accurate identification of L-2HG and D-2HG basing on the terahertz time-domain spectroscopy.

## Methods

### Experimental setup

A THz time-domain spectroscopy (THz-TDS) system was used^15^. In our experiments, 800 nm femtosecond laser characterized by pulse duration of 100 fs, repetition rate of 76 MHz, and average power of 150 mW was used. The emitted laser beam was separated into two beams—pump and probe (split ratio was 50:50). Pump beam modulated by optical chopper was focused on a Gallium Arsenide (GaAs) and then emitted THz wave. The diverging THz beam was collected and focused by paraboloidal mirrors to pass though biological samples, and then the probe beam was used to detect THz wave with samples’ physical properties by photoconductive antennas()^15–17^. For our experimental system, the effective bandwidth for the measured signals is from 0.2 to 2.5 THz, the spectrum resolution is better than 15 GHz, signal to noise radio (SNR) is larger than 1000:1. Besides, all the spectra were averaged 128 times to ensure a high SNR.

The absorbance of samples α(ω) are calculated by using the following equation:

1 $${\rm{\alpha }}({\rm{\omega }})=\,\mathrm{log}({{\rm{I}}}_{{\rm{ref}}}({\rm{\omega }})/{{\rm{I}}}_{{\rm{sam}}}({\rm{\omega }}))/{\rm{d}},$$

where d = 0.2 mm is the thickness of sample, I_sam_(ω) = E_sam_(ω) × E_sam_(ω)^*^ is the power spectrum of the sample, and I_ref_ (ω) = E_ref_ (ω) × E_ref_(ω)^*^ is the power spectrum of reference signal^15,18^.

### Samples preparation and measurement

The samples of L-2HG and D-2HG were purchased in powder (Sigma-Aldrich) with the highest purity available (≥98%). A sample device composed of two PE boards with thickness of 1 mm was used where samples were limited in a circular groove (thickness and diameter were all 0.2 mm). The entire THz spectroscopy system was enclosed in a sealed box filled with dry air (humidity <2%) to reduce the effects from water vapor.

## Results

### THz absorption spectra of 2HG isomers

**Figure 1:**
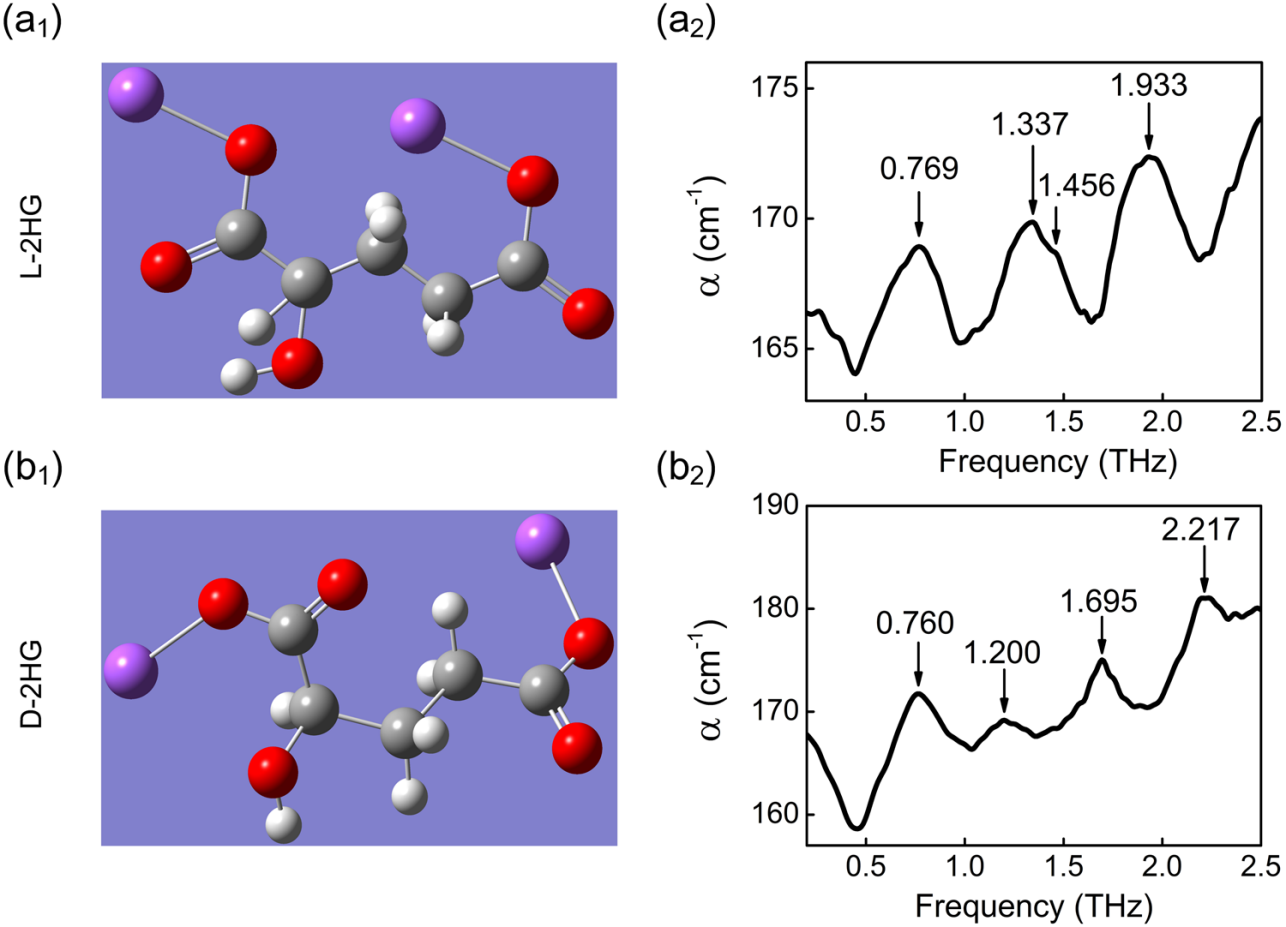
The molecular structure of (**a**_**1**_) L-2HG and (**b**_**1**_) D-2HG. THz spectrum of (**a**_**2**_) L-2HG and (**b**_**2**_) D-2HG. The corresponding THz absorption peaks are labeled on each figure.

The molecular structure and the corresponding THz absorption spectra of L-2HG and D-2HG are shown in Fig. 1. Clearly, as the isomers of 2HG, L-2HG and D-2HG are similar in molecular structure, which determines that their THz absorption spectra share certain characteristics. For example, the vibration peak at 0.769 THz of L-2HG corresponds well with 0.760 THz of D-2HG, where the difference is only 0.009 THz within the spectrum resolution. On the other side, their spectral differences still exist, including the shape of spectra and the positions of absorption peaks [see Fig. 1 and Table 1]. Specially, we notice that the peak at 1.456 THz has a very low amplitude but high repeatability, thereby it is still identified as one of absorption peaks.

**Table 1:** The list of absorption peaks of L-2HG and D-2HG.

L-2HG	Simulation	D-2HG	Simulation	Experimental deviation between L-2HG and D-2HG	Analysis
Frequency (THz)					
0.769	0.613	0.760	0.645	0.009	the torsion of the whole carbon chain
1.137	1.248	1.200	1.065	0.137	Vibration and aggressively torsion of carbon chain
1.456	1.680	1.695	1.835	0.239	torsion and vibration of the ring
1.933	2.066	2.217	2.354	0.284	concertina movement of the whole ring

### THz spectra calculated by Density Functional Theory

It has been known that THz absorption peaks are originated from the vibration and rotation of atoms and functional groups in the molecule. Different pattern arrangements between atoms—functional groups—directly determine the physical properties of substances and the corresponding THz absorption spectra. Therefore, isomers such as L-2HG and D-2HG present different spectral information as shown in Fig. 1. Based on this, we also want to know the correspondence between functional groups and THz absorption peaks. Therefore, a series of calculations were performed with Density Functional Theory (DFT) to study the vibrational and rotational modes of the L-2HG and D-2HG (B3LYP theory and 6–311 + G(d, p) basis set)^19–21^. The corresponding three-dimensional signal molecule structure model of L-2HG and D-2HG are obtained from ChemSpider website.

## Discussion

**Figure 2:**
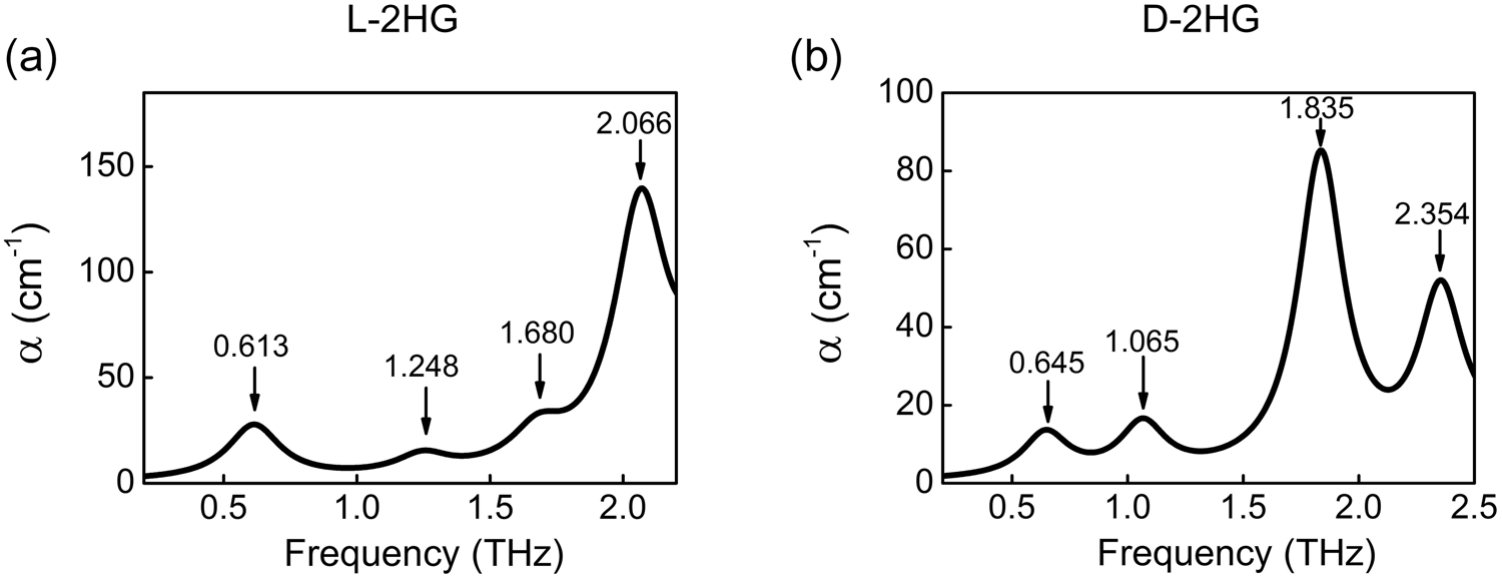
DFT spectral calculation results of (**a**) L-2HG and (**b**) D-2HG.

**Figure 3:**
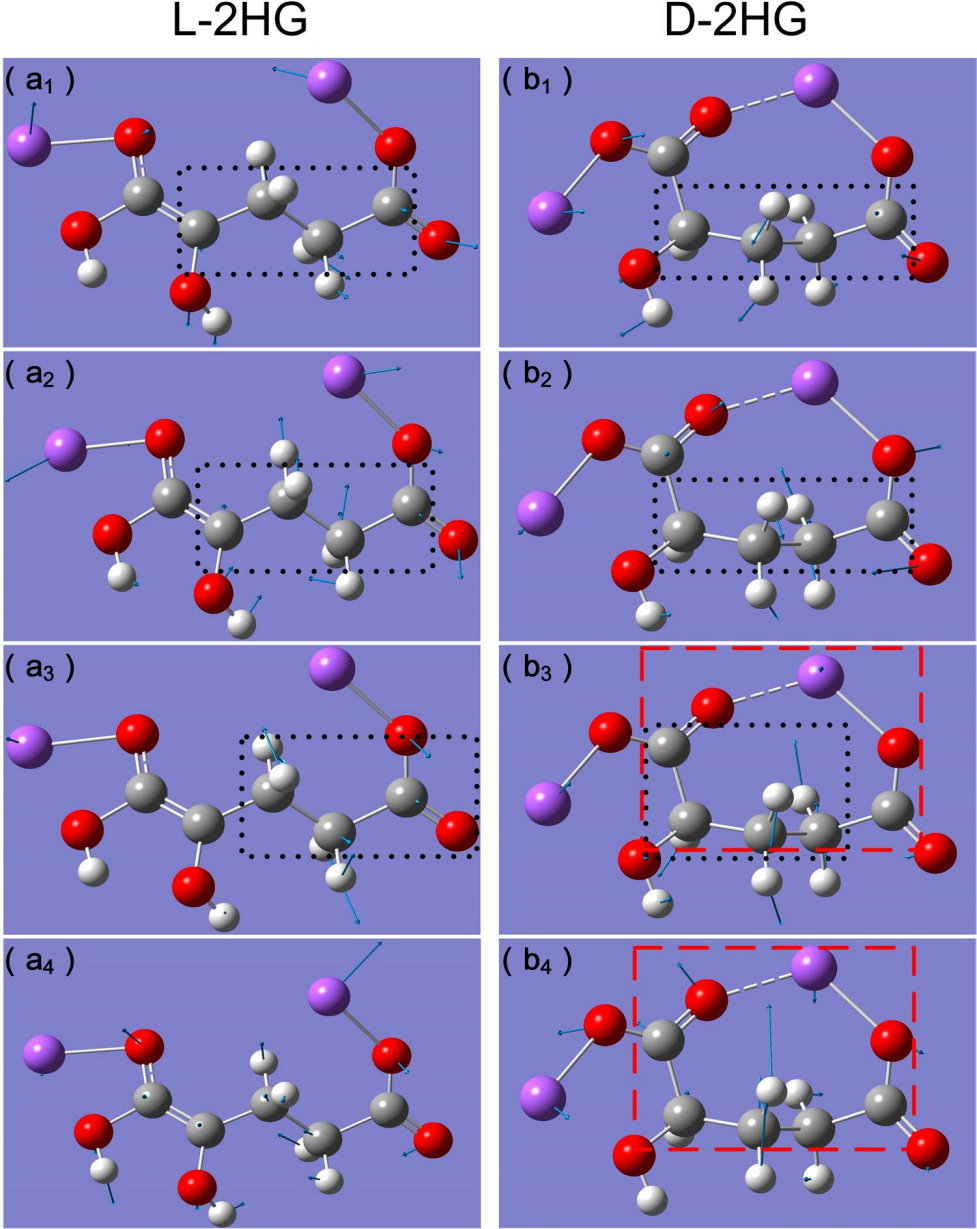
Vibration modes of absorption peaks of (**a**_**1**_ **–a**_**4**_) L-2HG and (**b**_**1**_ **–b**_**4**_) D-2HG. (**a**_**1**_) 0.769 THz (**a**_**2**_) 1.337 THz (**a**_**3**_) 1.456 THz (**a**_**4**_) 1.933 THz, (**b**_**1**_) 0.760 THz (**b**_**2**_) 1.200 THz (**b**_**3**_) 1.695 THz (**b**_**2**_) 2.217 THz. White, gray, red, and purple atoms represent H, C, O, Na atoms, respectively. Blue arrows indicate the vibrational direction of atom. Black dotted boxes refer to the dominant functional groups in the vibration modes. Red dashed boxes indicate the entire molecular vibrational ring.

Figure 2 presents the DFT simulation results of L-2HG and D-2HG, and the corresponding comparisons with experimental results are shown in Table 1. According to the results of theoretical calculation, we can determine the origin of these absorption peaks: firstly, as mentioned above, the vibration peak at 0.769 THz of L-2HG is highly similar to 0.760 THz of D-2HG, which is caused by their highly similar vibration mode—the torsion of the whole carbon chain dominated by the butyrate group [the black dotted boxes in Fig. 3(a_1_,b_1_)]. Figure 3(a_2_) shows that extensional motion of the whole carbon chain, based on the to-and-fro vibration of the butyrate group, causes the formation of absorption peaks at 1.337 THz of L-2HG. The vibration modes of D-2HG at 1.200 THz are not only include the formation factors of L-2HG at 1.337 THz, but also include the aggressively torsion of the butyrate group [see Fig. 3(b_2_)]. Briefly, the same dominant functional groups determine their THz absorption peaks are close to each other, while the different vibrational modes lead to the variation in the waveform and amplitude. On the contrary, when the dominant functional groups are different, the corresponding THz absorption peaks will have large differences. For example, the peak at 1.456 THz of L-2HG is caused by rotation of the propyl group [see Fig. 3(a_3_)], while the torsion and vibration of the ring affected by the butyrate group [see Fig. 3(b_3_)] forms the peak at 1.695 THz. In addition, comparing with the strong contractions of the whole ring resulted in the peak at 2.217 THz of D-2HG, the peak at 1.933 THz of L-2HG is completely caused by the up and down vibration of the whole carbon chain [see Fig. 3(a_4_,b_4_)].

**Figure 4:**
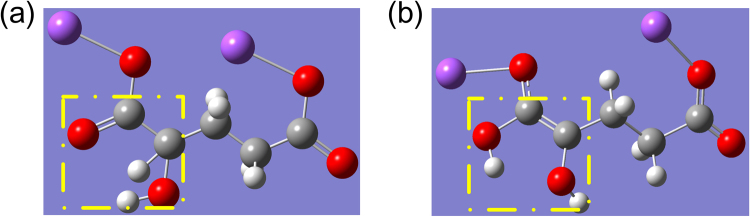
The molecular structure of L-2HG (**a**) before (**b**) after the structure optimization. The yellow dashed boxes indicate the transfer of the proton (hydrogen atom).

Furthermore, basing on the DFT analysis, we can deduce that the differences between isomers absorption peaks originates from the proton transfer (proton: hydrogen atom) in molecular structure [see Fig. 4]. In the case of L-2HG, it seems that during the process of molecular structure optimization, both the carbon hydrogen bond in the sodium hydroxyacetate group and the carbon oxygen double bonds are broken. As a result, two carbon atoms both have a lone pair electron, which then form a new carbon-carbon double bond to ensure the valence state of these carbon atoms. At the same time, the hydrogen atom combines with carbonyl oxygen to form a new hydroxyl, i.e., finish the process of proton transfer. While in the case of D-2HG, the carbon chain is “softer” than that of L-2HG, i.e., itself has a stable molecular vibrational ring, which determines no subsequent bond cleavage and the final proton transfer will happen.

## Conclusion

We experimentally demonstrated the use of THz-TDS for the measurement and identification of 2-HG isomers, i.e., L-2HG and D-2HG. Combing with the DFT function in Gaussian software, we calculated the THz absorption spectra and analyzed their corresponding relation with the molecular functional groups, which agree well with the experimental results. It is indicated that the great differences of the isomer between L-2HG and D-2HG are mainly attributed to their structures: the carbon chain of D-2HG is more flexible than L-2HG and it is easy to form a ring structure during vibration. While the vibration modes of L-2HG is the torsion and swing of carbon chain caused by the proton transfer. These results about THz identification of isomers have great meaning for the medicine identification and analysis, and also the further surgical operation and imaging.
